# Supplementary material for: T6SS4 is heterogeneously expressed in Yersinia pseudotuberculosis and is a target for transcriptional and post-transcriptional regulation
Source: PLoS Pathog. 2025 Sep 24;21(9):e1013356. doi: 10.1371/journal.ppat.1013356 (PMC12503267; doi:10.1371/journal.ppat.1013356)
Supplement: S1 Table — (DOCX) [file ppat.1013356.s001.docx]

**Table S 1: Bacterial strains and plasmids**

| **Strains, plasmids** | **Description** | **Source and reference** |
| --- | --- | --- |
|  |  |  |
| **Bacterial strains**  ***E. coli K12*** |  |  |
| SM10λ*pir* | *thi thr leu tonA lacY supE recA*::RP4-2-Tc::Mu Km λ*pir* | [1] |
| ***Y. pseudotuberculosis*** |  |  |
| YPIII | pIB1 | [2] |
| YP50 | YPIII, ∆*ymoA::kan^R^* (**∆*ymoA***) | [3] |
| YP53 | YPIII, ∆*csrA::kan^R^* (**∆*csrA***) | [4] |
| YP78 | YPIII, **∆*lon*** | This study |
| YP138 | YPIII, **∆*pnp*** | [5] |
| YP154 | YPIII, **∆*rovC*** | [6] |
| YP356 | YPIII, **∆*rnc*** | [7] |
| YP412 | YPIII, ***clpV4-gfp*** | [6] |
| YP417 | YPIII, ∆*csrA::kan^R^*, *clpV4-gfpmut3.1* (∆***csrA* *clpV4-gfp***) | [6] |
| YP474 | YPIII, ∆*ymoA::kan^R^, clpV4-gfpmut3.1* (∆***ymoA clpV4-gfp***) | This study |
| YP544 | YPIII, **∆*lon*, *clpV4-gfp*** | This study |
| YP566 | YPIII, Δ3,925,503-3,925,531 (intergenomic region between *hcp4* and *tssE4*) **(∆intergenomic region**) | This study |
|  |  |  |
| **Plasmids** |  |  |
| pBAD30 | Cloning vector, ori p15A, Amp^R^ | [8] |
| pAKH3 | pGP704, ori R6K, *sacB*^+^, Amp^R^ | [9] |
| pASS90 | pAKH3, YPK_3559 (*clpV4*)-*Ala-Gly-linker-gfpmut3.1* | [6] |
| pANK4 | pFU98, SC101*, 5’UTR*_rovC_*-*mCherry*  (-579 to +13)^a^, Cm^R^ | This study |
| pANK15 | pFU98, SC101*, 5’UTR_YPK_3566_-*gfp*  (-579 to +15)^a^, Cm^R^ | This study |
| pANK25 | pFU31, 5’UTR_YPK_3563_-*gfpmut3.1*  (-212 to +30)^a^, Amp^R^ | This study |
| pANK45 | pAKH3, 3,925,503-3,925,531 replaced with a 24 bp linker sequence | This study |
| pCP20 | Mutagenesis vector, FRT, ori CI857, Bla^R^, Cm^R^ | [10] |
| pCK56 | pAKH3, ∆*hcp4* (YPK_3563) | This study |
| pFU31 | colE1, *gfpmut3.1* | [11] |
| pKD4 | Mutagenesis vector R6K, FRT, Amp^R^, Kan^R^ | [12] |
| pVK25 | pBAD30, 5’UTR-*rovC* (-39)^a^, Amp^R^ | [6] |

^a^nucleotides relative to transcriptional start
^R^resistance casette

References

1. Simon R, Priefer U, Puhler A. A broad host range mobilization system for *in vivo* genetic engineering: transposon mutagenesis in gram negative bacteria.

2. Bölin I, Norlander L, Wolf-Watz H. Temperature-inducible outer membrane protein of *Yersinia pseudotuberculosis* and *Yersinia enterocolitica* is associated with the virulence plasmid. Infect Immun. 1982;37: 506–512. doi:10.1128/iai.37.2.506-512.1982

3. Böhme K, Steinmann R, Kortmann J, Seekircher S, Heroven AK, Berger E, et al. Concerted Actions of a Thermo-labile Regulator and a Unique Intergenic RNA Thermosensor Control *Yersinia* Virulence. Isberg RR, editor. PLoS Pathog. 2012;8: e1002518. doi:10.1371/journal.ppat.1002518

4. Heroven AK, Böhme K, Rohde M, Dersch P. A Csr-type regulatory system, including small non-coding RNAs, regulates the global virulence regulator RovA of *Yersinia pseudotuberculosis* through RovM. Mol Microbiol. 2008;68: 1179–1195. doi:10.1111/j.1365-2958.2008.06218.x

5. Kusmierek M, Hoßmann J, Witte R, Opitz W, Vollmer I, Volk M, et al. A bacterial secreted translocator hijacks riboregulators to control type III secretion in response to host cell contact. PLoS Pathog. 2019;15: e1007813. doi:10.1371/journal.ppat.1007813

6. Knittel V, Sadana P, Seekircher S, Stolle A-S, Körner B, Volk M, et al. RovC - a novel type of hexameric transcriptional activator promoting type VI secretion gene expression. Mecsas J, editor. PLoS Pathog. 2020;16: e1008552. doi:10.1371/journal.ppat.1008552

7. Meyer I, Volk M, Salto I, Moesser T, Chaoprasid P, Herbrüggen A-S, et al. RNase-mediated reprogramming of *Yersinia* virulence. Isberg RR, editor. PLoS Pathog. 2024;20: e1011965. doi:10.1371/journal.ppat.1011965

8. Guzman LM, Belin D, Carson MJ, Beckwith J. Tight regulation, modulation, and high-level expression by vectors containing the arabinose PBAD promoter. J Bacteriol. 1995;177: 4121–4130. doi:10.1128/jb.177.14.4121-4130.1995

9. Heroven AK, Sest M, Pisano F, Scheb-Wetzel M, Steinmann R, Böhme K, et al. Crp Induces Switching of the CsrB and CsrC RNAs in Yersinia pseudotuberculosis and Links Nutritional Status to Virulence. Front Cell Inf Microbio. 2012;2. doi:10.3389/fcimb.2012.00158

10. Cherepanov PP, Wackernagel W. Gene disruption in *Escherichia coli*: TcR and KmR cassettes with the option of Flp-catalyzed excision of the antibiotic-resistance determinant. Gene. 1995;158: 9–14. doi:10.1016/0378-1119(95)00193-A

11. Uliczka F, Pisano F, Kochut A, Opitz W, Herbst K, Stolz T, et al. Monitoring of Gene Expression in Bacteria during Infections Using an Adaptable Set of Bioluminescent, Fluorescent and Colorigenic Fusion Vectors. Abu Kwaik Y, editor. PLoS ONE. 2011;6: e20425. doi:10.1371/journal.pone.0020425

12. Datsenko KA, Wanner BL. One-step inactivation of chromosomal genes in *Escherichia coli* K-12 using PCR products. Proc Natl Acad Sci USA. 2000;97: 6640–6645. doi:10.1073/pnas.120163297
