# Supplementary material for: T6SS4 is heterogeneously expressed in Yersinia pseudotuberculosis and is a target for transcriptional and post-transcriptional regulation
Source: PLoS Pathog. 2025 Sep 24;21(9):e1013356. doi: 10.1371/journal.ppat.1013356 (PMC12503267; doi:10.1371/journal.ppat.1013356)
Supplement: S2 Table — (DOCX) [file ppat.1013356.s002.docx]

**Table S 2: Oligonucleotides for DNA amplification**

| **Primer** | **Sequence (5‘-3‘)** | **Description** |
| --- | --- | --- |
| I38 | cctcgcattgttatcgttg | fw for ∆*lon* mutant control PCR |
| I39 | cttcttgcgatccgtcac | fw for ∆*lon* mutant control PCR |
| I407 | cacgagatttccagtaatctggcggaagctaaactaagagag agctcgtgtaggctggagctgcttc | fw for ∆*lon* mutagenesis, *kan*^R^ region at 3'-end (underlined) |
| I408 | ggcgctactggctgggcaccaaaggccgggtgttccaacgca atggccatatgaatatcctccttagt | rev for ∆*lon* mutagenesis, *kan*^R^ region at 3'-end (underlined) |
| III393 | ccgacgtaaagccgcgatac | qRT-PCR *sopB* control fw |
| III394 | cctcgttcataagcactcgtc | qRT-PCR *sopB* control rev |
| IV94 | ctgaattaatccaatcagagctgg | qRT-PCR YPK_3567 (*rovC*) fw |
| IV95 | ccagtattactgagtttaactctaac | qRT-PCR YPK_3567 (*rovC*) rev |
| IX045 | agagcaattaagcctcgcc | qRT-PCR YPK_3565 (*vipA4*) fw |
| IX046 | tgcctcaagcagtttttcc | qRT-PCR YPK_3565 (*vipA4*) rev |
| IX687 | tcccgggtacctgcaggaatatttgcccttataaa | fw pANK4 fragment 1 |
| IX688 | gaaagaagcgtttccaagggcgaggaggataacatg | rev pANK4 fragment 1 |
| IX689 | gaaagaagcgtttccaagggcgaggaggataacatg | fw pANK4 fragment 2 |
| IX690 | ggatgagctgtacaaataaggccgctctagaggcat | rev pANK4 fragment 1 |
| V649 | gctcaccttacgtgccagcgt | qRT-PCR YPK_3552 (*tssK4*) fw |
| V650 | ccgcattatcgatccaccctatg | qRT-PCR YPK_3552 (*tssK4*) rev |
| V651 | cggcccaactggatgtgctc | qRT-PCR YPK_3559 (*clpV4*) fw |
| V652 | catgcagatggcggctttgc | qRT-PCR YPK_3559 (*clpV4*) rev |
| V653 | catcttcgacattatttttaactgtc | qRT-PCR YPK_3566 (*tssA4*) fw |
| V654 | gttcacaatgcagttggtaactc | qRT-PCR YPK_3566 (*tssA4*) rev |
| VII773 | gcagctttagtcgattactttc | Northern blot *hcp4* probe fw |
| VII774 | cggttgttcaatttcagatcg | Northern blot *hcp4* probe rev |
| X108 | aggatcccgggtacctgcagatctatgtcctcttattttg | fw pANK15 fragment 1 |
| X109 | ataatatgagtgacatatttgcggcggccgcaggaggacgt aaaggagaagaacttttca | rev pANK15 fragment 1 |
| X110 | ataatatgagtgacatatttgcggcggccgcaggaggacgt aaaggagaagaacttttca | fw pANK15 fragment 2 |
| X111 | tggatgaactatacaaataacggccgctctagaggcatca | rev pANK15 fragment 2 |
| X223 | aggtggtggtggtggttc | qRT-PCR YPK_3563 (*hcp4*) fw |
| X224 | tgaccttgacctgatttacgac | qRT-PCR YPK_3563 (*hcp4*) rev |
| X382 | ttctcctttacgcatgtcgactcgagccgcaagcat gctgaaatg | rev pANK25 fragment 1 |
| X383 | aggatcccgggtacctgcagccatccaacaaggaaa gcga | fw pANK25 fragment 1 |
| X558 | tcttctagaggtaccgcatgccgcgctcacttctatttac | fw pANK45 fragment 1 |
| X559 | aactcgagccgcaagcatgctgaactaatgcatttctgactt | rev pANK45 fragment 2 |
| X560 | ttcagcatgcttgcggctcgagttttccaaactggattgggg | fw pANK45 fragment 2 |
| X561 | caatttgtggaattcccggtccggaaccatggcaatgg | rev pANK45 fragment 2 |

fw = forward, rev = reversed
