# Supplementary figures and images for: T6SS4 is heterogeneously expressed in Yersinia pseudotuberculosis and is a target for transcriptional and post-transcriptional regulation
Source: PLoS Pathog. 2025 Sep 24;21(9):e1013356. doi: 10.1371/journal.ppat.1013356 (PMC12503267; doi:10.1371/journal.ppat.1013356)

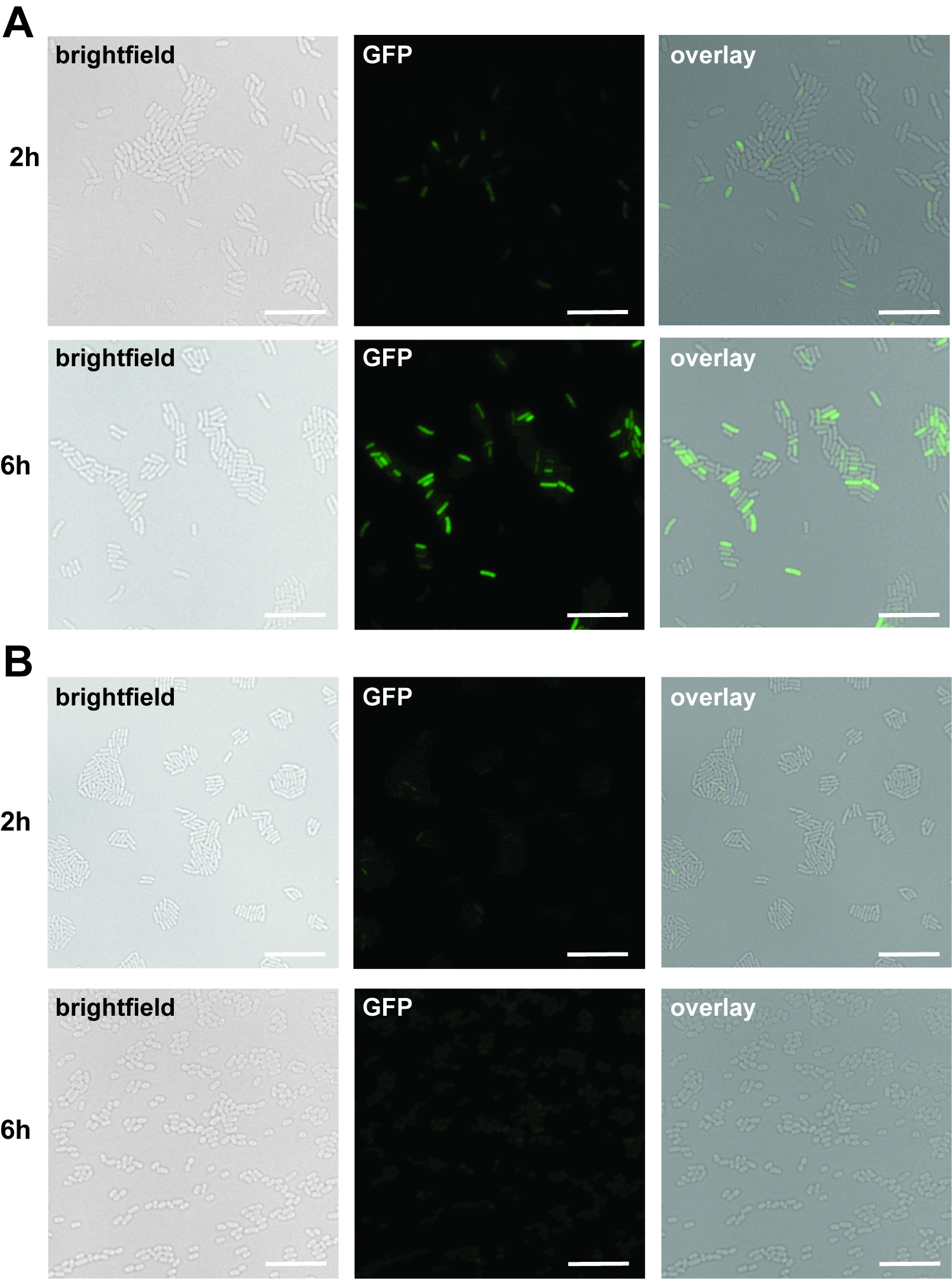

Supplement: S1 Fig — Fluorescence microscopy of Y. pseudotuberculosis wt clpV4-gfp incubated for two and 6 h at 25°C (A) or 37°C (B). Representative images of the brightfield and GFP channels and an overlay of both channels were shown. Bacteria were imaged on agarose pads containing 1% agarose, and the scale bar represents 10 µm. (TIF) [file ppat.1013356.s003.tif]

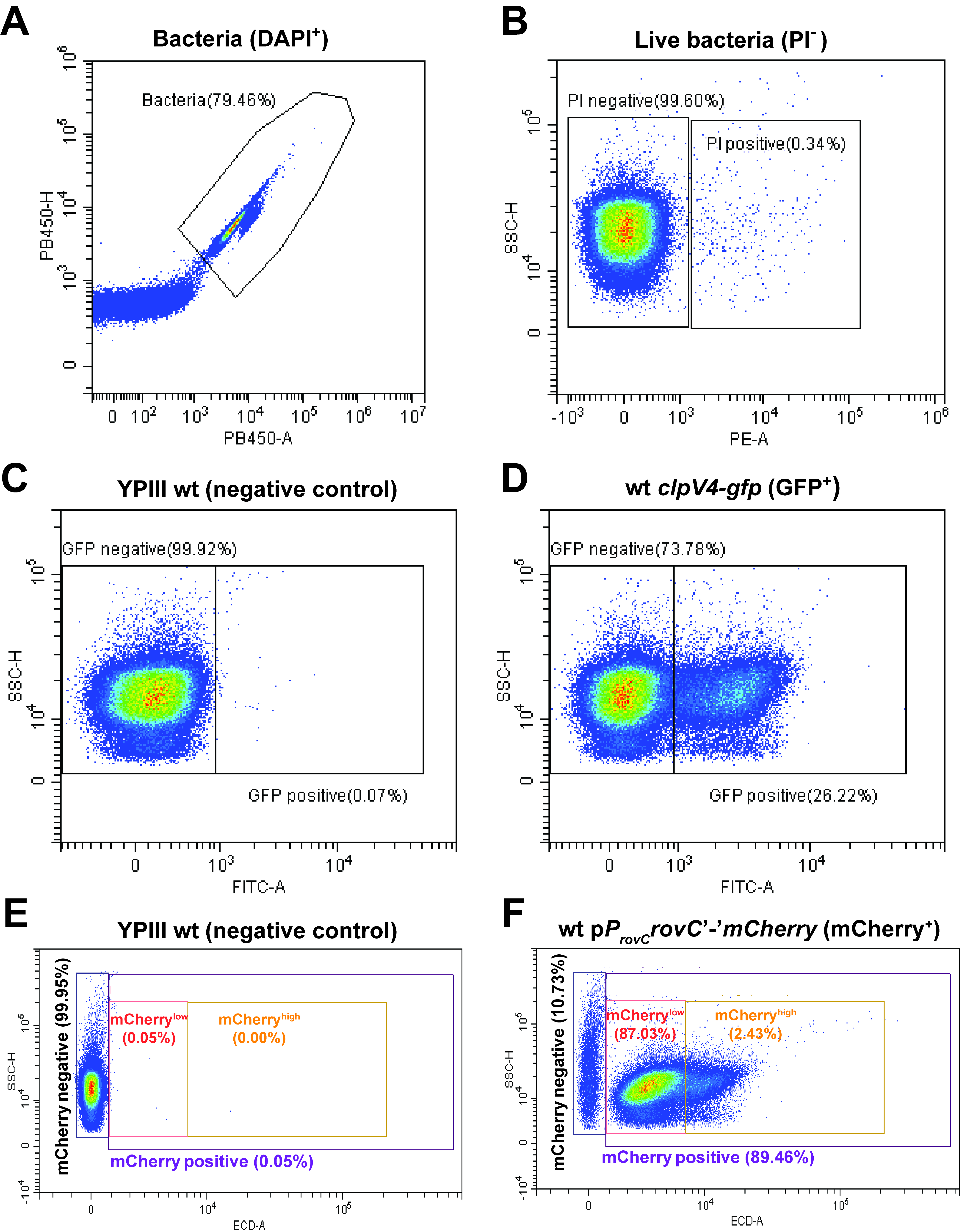

Supplement: S2 Fig — (A) Samples were stained with DAPI to gate for bacteria. (B) PI staining to exclude dead bacteria. (C) Negative control (YPIII wildtype) for GFP-expressing bacteria. (D) Gate for GFP-expressing bacteria. (E) Negative control (YPIII wildtype) for mCherry-expressing bacteria. (F) Gates for mCherry-expressing bacteria, divided into low and high expression intensity. H = height, A = area, SSC = side scatter. Exemplary plots are shown. (TIF) [file ppat.1013356.s004.tif]

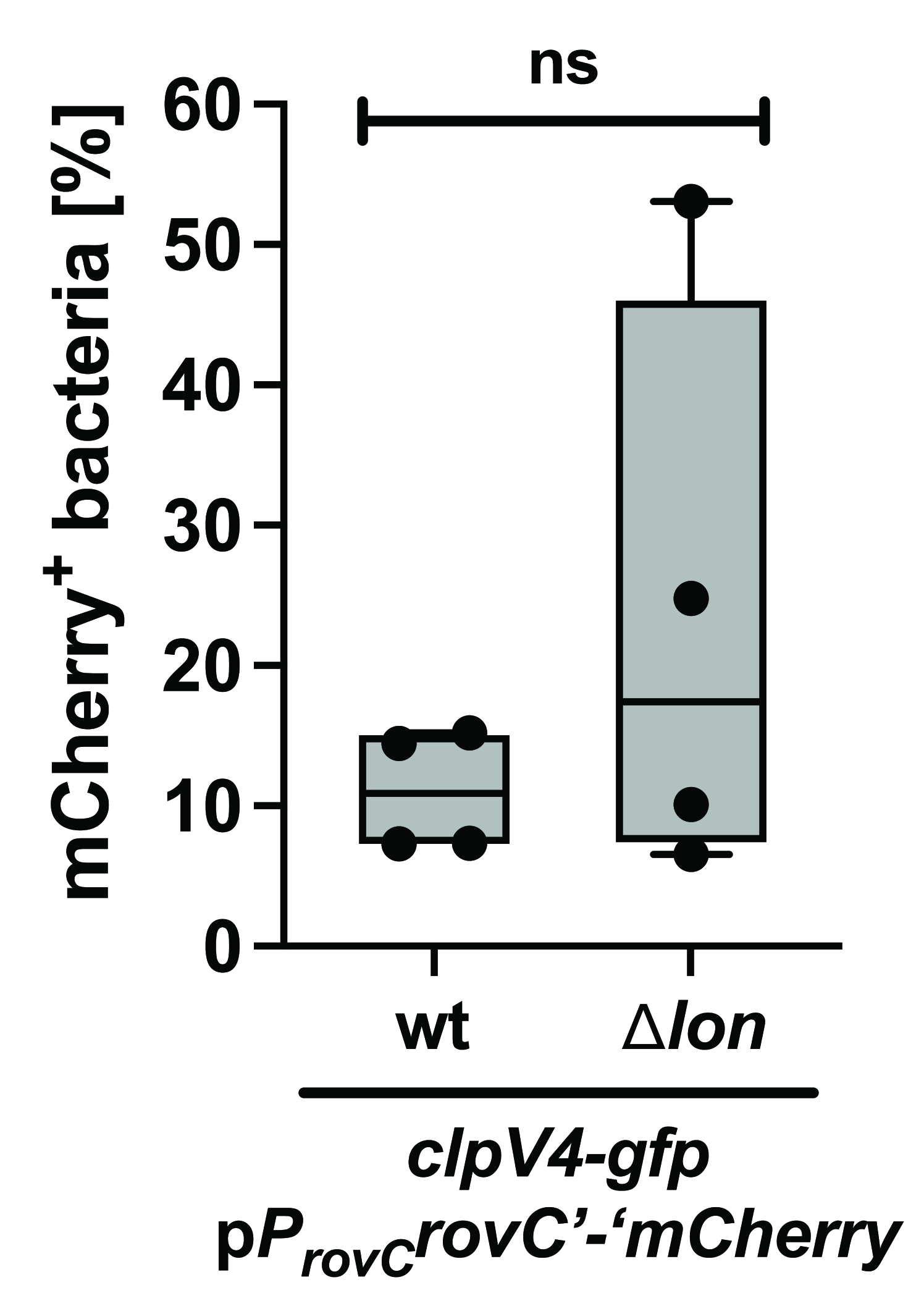

Supplement: S3 Fig — Wt clpV4-gfp pProvC rovC’-‘mCherry and ∆lon clpV4-gfp p pProvC rovC’-‘mCherry were incubated overnight at 25°C. 1 x 10^5^ bacteria were analyzed with flow cytometry, and experiments were performed in four independent experiments. Statistical significance was tested with an unpaired t-test. ns = not significant p > 0.05. (TIF) [file ppat.1013356.s005.tif]

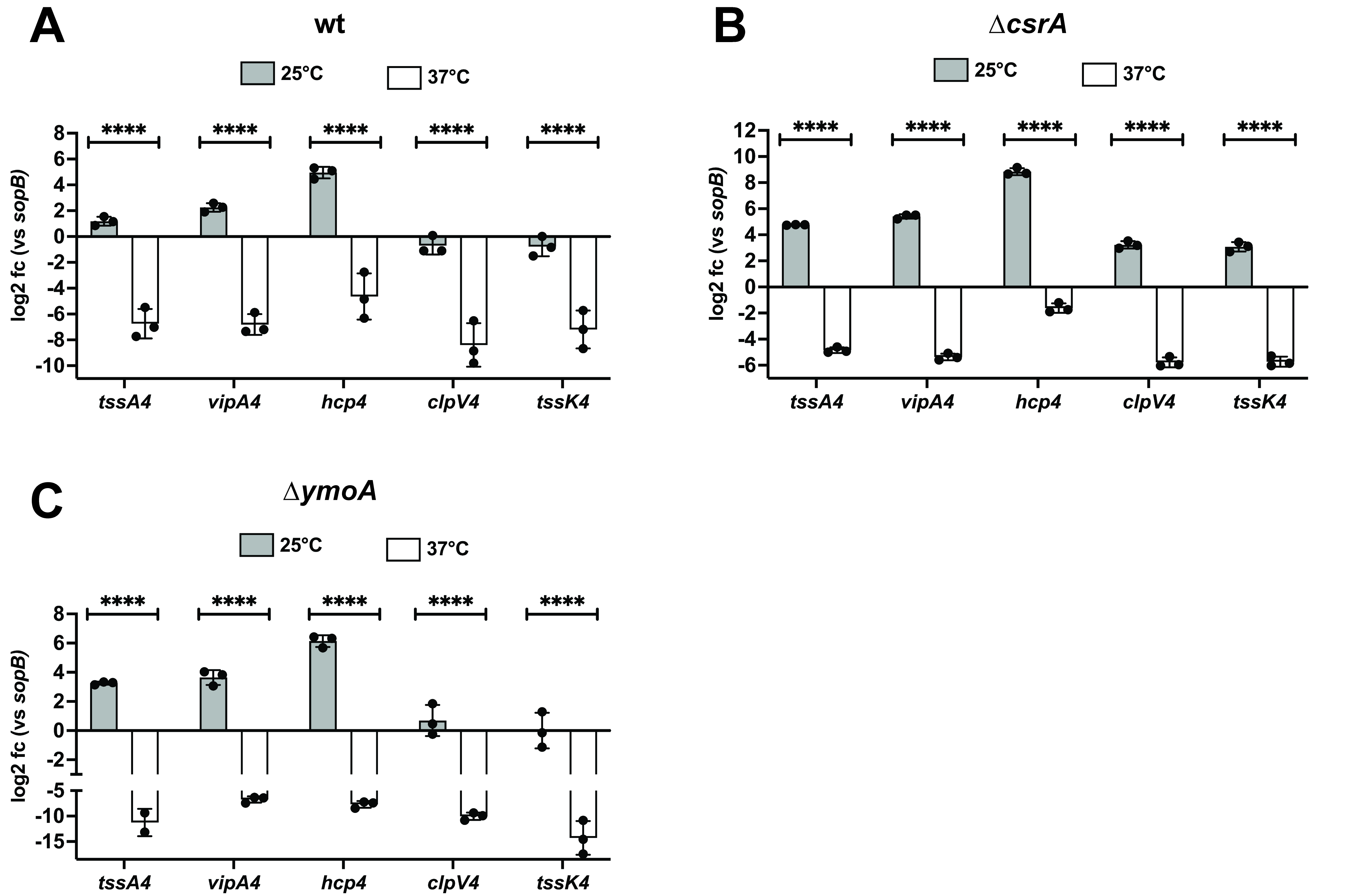

Supplement: S4 Fig — (A-C) Total RNA of an overnight culture of wt, ∆csrA, and ∆ymoA was isolated to perform qRT-PCR. Specific primer pairs for five T6SS4 genes were used to determine the expression within the T6SS4 operon. Log2-fold changes were calculated between the T6SS4 transcript and sopB as a non-temperature-regulated reference gene [6]. Experiments were performed in three biological replicates, and significant differences were determined using a Two-Way ANOVA with Šidák correction **** = p ≤ 0.0001. (TIF) [file ppat.1013356.s006.tif]

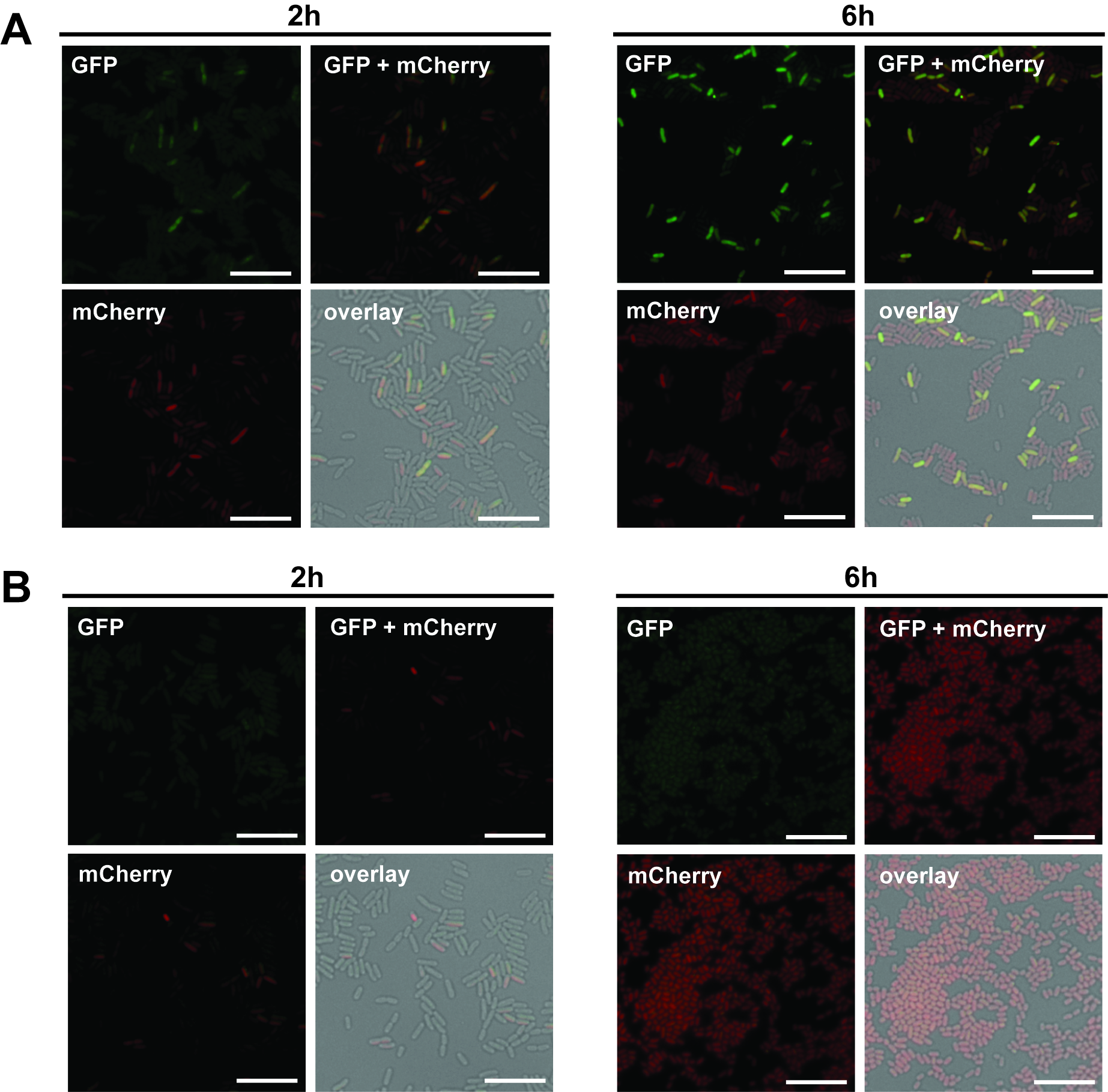

Supplement: S5 Fig — Fluorescence microscopy of Y. pseudotuberculosis wt clpV4-gfp harboring pProvCrovC’-‘mCherry. Strains were incubated for 2 h and 6 h at either 25°C (A) or 37°C (B). Representative images of the GFP and mCherry channels, overlays of the GFP and mCherry channels, and an overlay of all channels with the brightfield, were shown. Bacteria were imaged on agarose pads containing 1% agarose, and the scale bar represents 10 µm. (TIF) [file ppat.1013356.s007.tif]

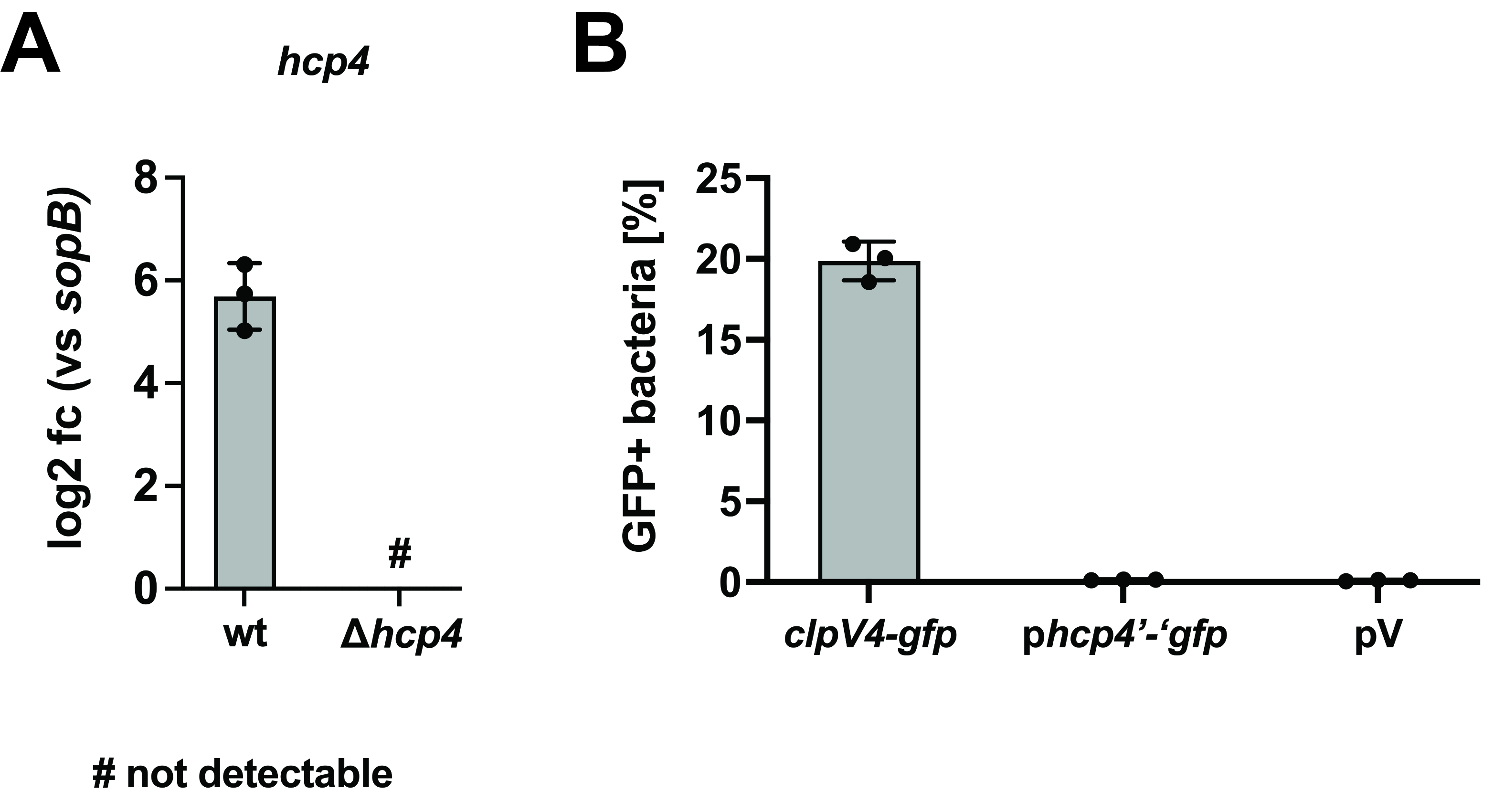

Supplement: S6 Fig — A low copy plasmid harboring the upstream region of hcp4, phcp4’-‘gfp (-212 to +30 base pairs with respect to the translational start site) was transformed into YPIII wt. Samples were incubated in 20 ml LB overnight at 25°C. wt clpV4-gfp was used as a control to compare the amount of clpV4-gfp expressing bacteria to the potential hcp4 promoter activity. Samples were taken for flow cytometry, and 1 x 10^5^ cells were analyzed. Experiments were performed in three biological replicates. The data depict the mean and standard deviation. pV = empty vector control. (TIF) [file ppat.1013356.s008.tif]

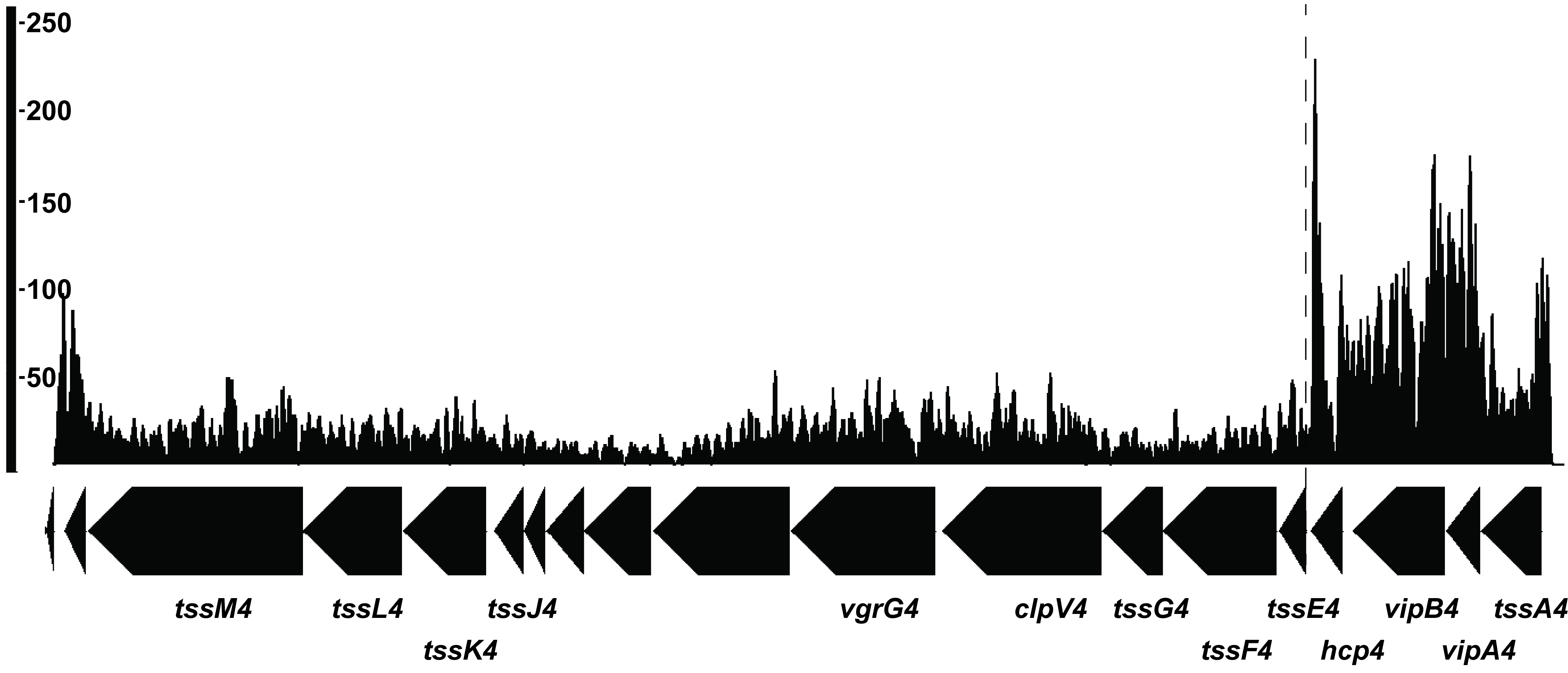

Supplement: S7 Fig — RNA coverage of all T6SS4 genes from samples of YPIII wt, incubated for 2 h at 25°C. Data was taken from Meyer et al., 2024 [46]. (TIF) [file ppat.1013356.s009.tif]
